# Measuring childhood socioeconomic position in health research: Development and validation of childhood socioeconomic position questionnaire using mixed method approach

**DOI:** 10.15171/hpp.2019.05

**Published:** 2019-01-23

**Authors:** Uma Vadassery Sankar, V Raman Kutty, TN Anand

**Affiliations:** ^1^Achutha Menon Centre for Health Science Studies, Sree Chitra Tirunal Institute for Medical Science and Technology, Trivandrum, Kerala, India; ^2^Research Fellow, Health Action by People, Thiruvananathapuram, Kerala, India

**Keywords:** Childhood socioeconomic position, Questionnaire, Psychometric properties, Validation, Measurement, Development

## Abstract

**Background:** There is no single best indicator to assess the childhood socioeconomic position (CSEP) in public health research. The aim of the study is to develop and validate a new questionnaire, with adequate psychometric properties, to measure the childhood SEP of the young adults.

** Methods:** The first phase consisted of a qualitative phase to identify the variables to measure childhood SEP through the in-depth interviews among 15 young adults (18-45 years) of rural Kerala. The second phase was a quantitative phase to validate the questionnaire through a cross sectional survey among 200 young adults of Kerala. We did content validity, reliability tests and construct validity by using exploratory factor analysis of the questionnaire to demonstrate its psychometric properties.

** Results:** The qualitative analysis reported 26 variables spread across 5 domains to measure the CSEP. Finally, the questionnaire has 11 questions with 3 domains named as value added through paternity, maternal occupation-related factors and parental education. The questionnaire has good reliability (Cronbach's α=0.88) also.

**Conclusion:** We have developed a reliable and valid questionnaire to measure the childhood SEP of younger adults and can be used in various public health research.

## Introduction

Socioeconomic position (SEP) during childhood is associated with each increase on the SEP hierarchy associated with an increase in health benefits.^1^ The link between childhood SEP (CSEP) and chronic disease are explained in various life course models like the critical period model, pathway model and accumulation of risk models.^2^ Life course models considered the childhood SEP as the critical period influencing the early and late life biological and social exposures and its direct and indirect effect on developing chronic illness in the adult life.^3^ These observations conclude the CSEP as an independent predictor of various adult health outcomes (both physical and mental).^1-4^ CSEP decides the early exposure of various risk factors of non-communicable diseases (NCDs) to an individual and it creates various biological and psychosocial mechanism for the development of NCDs at an early age of an individual^5^.

### Operationalization of the construct ‘childhood socioeconomic position’ 

CSEP can be observed as the interface between the family’s attempt to position themselves in society and the society’s attempt to position the family in its context.^4,5^ It is operationalized based on the 2 relevant theories on social stratification, Weber’s theory on SEP and Wright’s theory on social stratification. Individual is a unit of the family. So for a child, parents are the family member’s to create their own life chances through trading their job skills, educational qualification and attributes in a marketplace for social advantage.^5^ Parent’s attained level of education, occupation and possession of lands or other valuable materials are some examples of created life chances for a child.^6^ Wright’s theory of social stratification provides an idea of how the society positions an individual (unit of the family). That is based on his or her possession of skills, expertise and his or her relationship with authority. Educational qualification, job position and grade of occupation are some of the indicators used by society to decide the position of the individual. The interface between how the society positions an individual (Wright’s theory of social stratification) and the individual herself/himself achieves a position in the society (Weber’s theory of social stratification) is the basis for operationalizing to construct of ‘SEP’.^5-7^ An individual who studied in an elite school, one recruited by a multinational company or one from a disadvantaged group who nevertheless gets a government job through a quota system may have different positions in society.^8^

### Indicators of childhood socioeconomic position 

CSEP was measured retrospectively by using the indicators of parent’s occupation, education and family income.^5-8^ These indicators are also used as the indicators of economic stability and wealth during childhood period.^8^ Other indicators of CSEP included public housing, housing conditions and house ownership in a Swedish life course epidemiologic study.^9^ Most of the available childhood SEP indicators are more evident and measurable in developed countries, which is not relevant in the context of developing countries.^10^ Notably a new instrument to assess the childhood SEP would be useful for knowing the mechanism involved in the development of diseases (by knowing the exposure of various risk factors only for a particular group) especially in the NCD epidemiology and to help standardize future research in third world economies.

### Study aims

This paper reports on the development and validation of self-administered measure of CSEP of younger adults (18-45 years) based on a mixed-methods approach.

## Materials and Methods

**Figure 1:**
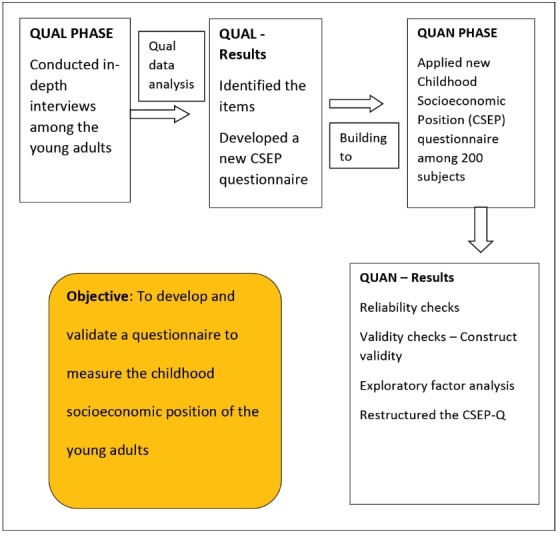
Sequential exploratory mixed method study.

This study follows sequential exploratory mixed method design (Figure 1). Here we begin the qualitative interview for exploratory purposes to identify the indicators of childhood SEP (phase 1) and follow up with a quantitative study for the purpose of validation of the newly developed questionnaire in a large sample (phase 2).

### Phase 1: Development of childhood socioeconomic position questionnaire 

The first step of this phase is to define the construct ‘CSEP’ through following 2 methods 1) literature review 2) experts panel review. We reviewed the available definitions of CSEP in the conceptual papers on SEP and health research studies. The search was made by following PRISMA guidelines. We searched PubMed and Google Scholar with no restrictions imposed on year of publication. Earliest age of recall of childhood events by an individual is 4 years as per the life course epidemiologic research.^11^ Younger age group can easily recall the childhood events and that ability will have there at a minimum age of 45 years and the adolescent will be end at the age of 18 years and initiated the process for official job at 25 years was the reason to keep the sample at 25-45 years. Expert panel of the study consist of an epidemiologist, a demographer, a social scientist, a sociologist, an economist, a general physician and a medical ethics specialist. The consensus was reached on the definition after repeated meeting with members of the expert panel. The definition of CSEP is the position of an individual at his/her childhood period in an economically and socially stratified society.^12^

*Research process*

As the knowledge of CSEP was limited, in-depth interviews were chosen as an appropriate data collection technique given the short time frame. We used interview guidelines for the prompting, reminding the necessary topics to cover, questions to ask and areas to probe. We conducted 2 freewheeling interviews with 1 male government employee and 1 female homemaker of rural Kerala. These interviews enabled us to identify keywords about the CSEP which served as the pointer of status and position in Malayali (That native language was Malayalam) society. Aided by these freewheeling interviews, we developed an interview guide with a set of open-ended questions. The questions related to (*i*) the participants understanding of socio-economic position specifically about the CSEP, (*ii*) the existence of different socioeconomic groups in their childhood period, and (*iii*) recognizing the different socioeconomic groups by considering their lifestyle, household assets, occupational group. A heterogeneous sample of 15 younger adults was interviewed for the qualitative phase of the study. The diversity of the sample was ensured for age, sex, occupation, place of living and socioeconomic status.

*Interview process*

We followed in-depth interview guideline for 12 interviews. Investigator used the life history technique for the remaining 3 interviews to assess the socioeconomic background of the individual with detailed description of the objects/variables with socioeconomic meanings. The stories appeared to work as a guide to identify the variables to measure the CSEP. This study used ‘itinerary’ method of data collection during the interview. Investigator was constantly going back to the socioeconomic meanings of the objects being listed (itinerary) and its interpretation in the society at various time periods. The process of moving from the general to particular was gradual.

*Procedure and analysis*

Meantime for the interview was 1 hour and 15 minutes. We used constant comparative method, where the analyst begins the analysis with the first data collected and constantly compares indicators, concepts and categories. In line with qualitative research methodology, data collection and analysis were concurrent. Categories were systematically compared and grouped into themes or major categories in selective coding. We have completed 15 interviews and after that, data saturation was reached as no new themes emerged. The coding assignments were reviewed and differences were resolved through discussion and consensus with 2 independent reviewers. Language equivalency and sense of integrity of the transcript was checked by 2 independent reviewers. We carried out the data analysis at three levels: open, axial and selective coding. Open coding was a line-by-line scrutiny of the data, to identify the codes expressed by the participants. Forty-eight codes in childhood SEP emerged from the data. We labelled related codes and grouped them into categories in axial coding. The next step was to identify a core category, which related to all other categories at the selective coding stage.

*Formatting the questionnaire*

All the codes are becoming variables. These variables were converted into questions. We have selected the Options for each question from the verbal transcript itself. There were 48 questions in the childhood SEP questionnaire. The questionnaire consists of both dichotomous yes/no questions and multiple choice questions. We blinded the interviewee about the scores of each option of the question and selected simple wording for each question to ensure the acquiescence of the questionnaire.

*Cognitive piloting*

Cognitive piloting is a method for identifying problems with question wording, comprehension, and recall and for ensuring that items are capturing the underlying construct. We used active probing approach (read question and probe responses), in cognitive piloting phase by asking questions like what made you say that? What does that mean to you? Please tell me what I was asking in your own words? To the respondents while reading each question by the researcher. We selected a purposive sample of 5 individuals between 25 and 45 years, with different occupation and socioeconomic profiles. We assessed the acceptability of the questionnaire, the time for completion, and the logical organization (both sequence and order) of the questions. After this, we tested the questionnaire on a sample of people representative of the target population. Throughout this process, we got written comments on the questionnaire by the participants. The words and interpretation of the questions are evaluated in pilot testing to refine the measurement tool.

### Phase 2: Validation of the childhood socioeconomic position questionnaire

The objective of this phase was to validate the CSEP questionnaire among the younger adults between the age group of 25-45 years. We checked the Content validity, reliability checks and the construct validity of the questionnaire in this phase.

*Content validity*

We established the content validity of the tool by assessing the content validity index. It depends on the professional subjective agreement on the relevance of each item in the tool by the same expert panel available for the developmental stage. The experts then rated each item on a 4-point scale. 1- Irrelevant, 2 - partially relevant, 3 - relevant and 4 - very relevant. The content validity index (CVI) is computed as the number of experts giving a rating of either 3 or 4 (thus dichotomizing the ordinal scale into relevant and not relevant), divided by the total number of experts. The items had CVI over 0.80 remained and the rest were discarded. The remaining items, which are very relevant to the topic, were modified, based on the experts’ opinions.

*Ranking the options*

We asked the respondent to rank the options under each question and to compare items to each other by placing them in order of their understanding about the SEP hierarchy existing at the time of childhood living (their childhood period). The validation survey participants did not know about the ranking order. The equal interval was assumed between the options (For example method of commuting to school (*a*) by school bus (*b*) by a private vehicle (*c*) by own bicycle (*d*) by walk).

*Reliability*

*Participants and procedure*

We selected 200 young adults (between 25-45 years) with different occupational background to meet the participant variable ratio 1:8 checking^8^ the validity measures of the questionnaire purposively from Three districts of Kerala (Kannur from North Kerala, Palakkad from Central Kerala and Trivandrum from Southern Kerala) to allow the heterogeneity in the socioeconomic profiles and geographic variation. The age group was maintained as between 25-45 years due to the demand of the draft questionnaire to use individual’s cognitive skills to reconstruct the memories about childhood period and its events (Table 1). Studies suggested that negligible loss of childhood memories will be there till the age of 45 years.^6^ This Sampling technique was purposive sampling i.e. purposely handpicking individuals from the population-based on the convenience of the researcher. Subjects completed questionnaires on daytime during their normal work hours in a semi-private area to minimize group interaction.

An individual item analysis was analysed by calculating the corrected item-total correlations (i.e. correlation between the total score and item score). Internal consistency is typically equated with Cronbach alpha. It is implicitly assumed that the average correlation of a set of items is an accurate estimate of the average correlation of all items that pertain to a certain construct. The calculated internal consistency for the variables was high with Cronbach alpha >0.70. If the items measure the same underlying concept than each item should correlate with the total score from the questionnaire or domain. This score can be biased, especially in the small sample sizes, as the item itself is included in the total score. So we calculated a corrected item-total correlation of the questionnaire. This removes the score from the item from the total score from the questionnaire or domain prior to the correlation. Kline^13^ recommends deleting any questionnaire item with a corrected item-total correlation of <0.3. There is no scope for doing test-retest reliability since the questionnaire collected the factual information.

*Construct validity*

We did an exploratory factor analysis to identify the complex interrelationship among the items and group of items that are part of a unified concept. We had no a priori assumptions about the relationships among factors. The maximum likelihood method was used, and the rotated matrix was extracted with direct oblimin rotation. The number of factors was decided using Kaiser’s criterion, which requires eigenvalues greater than one, in addition to Cattell’s scree plot test on the sedimentation graph. We did the factor loading only for those variables, whose value is more than 0.50 (Haier’s criteria for factor loading). Scree plot provides the visual diagram of the total variance associated with each factor. The gradual trailing off (scree plot) shows the rest of the factors usually lower than an Eigenvalue of 1.

## Results

### Qualitative phase: Development of childhood socioeconomic position questionnaire

Society considers the status of the overall family to decide a child’s SEP. A child from a rich family is considered rich and has an access to all kinds of facilities matched with their social status. All these indicators are interrelated with each other.

“*Society decides the socioeconomic position of a child by assessing their family’s social position. This social position is decided based on their caste, occupation and land assets hold by the head of the household. Father is the head of the household in a normal Malayalee family. If one kid is born in a rich family, we would call the rich kid as the child born with a gold spoon in the mouth (‘Swarna Karandi’). Society positions him based on the attained education, occupation and way of living if that child grew up and became an adult*.”

### Parental factors

Accessible and available facilities to a child begin from the intrauterine period itself. The first domain describes the factors related to parents. All the identified properties (items) in the parental domain are either endowed or decided by parents. Participants are of the opinion that *“parent’s occupation decides the socioeconomic level of a child. Father is the main breadwinner of the family. If the father and mother are government servants, their children belong to upper class…. People also consider the land assets to assess the socioeconomic background of an individual.*”

Permanent source of income for either or both of the parents (having any kind of monthly monetary benefits from the job which will continue until their death, even after the completion of their formal employment period), whether they are working in private or government sector, job position, parent’s education, total area of land (on an average) possessed, the caste they belong to and the place of living during their childhood (urban/rural) are the major properties (items) under the parental domain.

*“Most of the upper-class children belong to ‘upper’ caste. Muslims are poor. Thenumber of kids in the upper-class family will be less. 2 or 3 kids. But a number of kids in the lower family will be huge in number. Eight or nine.”*

*“Most families, the male member is the breadwinner. Only a few families have female breadwinners. Financial dependence was very common among the households. Male member will decide the allocation of financial resources and find the way to satisfy both major and minor financial needs of the family members.”*

### Gender 

Gender of a child is one of the important domains to measure childhood SEP. It decides the allocation of resources within a household. It decides the facilities available to a child during the childhood period. Parents are more likely to spend more money for the higher education of a boy. Parents invest more money for a girl child’s marriage instead of her education. A girl child in a poor family should learn all the household activities from her childhood period. But a boy child has the freedom to play outside, making more friendships and very limited restrictions to wander and enjoy the social ceremonies during the growing period. Gender is the second domain to measure the childhood SEP.

“*Our society gave more freedom to a boy child when compared to a girl child. A poor girl child learns all kinds of household activities especially brooming, mopping the floor, washing clothes, and preparing fish etc. It is considered as an offence by the parents if their boy child is doing all the above-mentioned household activities. Girl child always shows the hospitality and caring attitude towards the male members and elderly members of the family. Boy child always shows endowed behaviors of masculinity. They can roam anywhere in the neighbourhood. They can attend ‘pooram’ (a social celebration attached to a temple) or ‘perunnal’ (a social celebration attached to the church) etc. Family members encourage and accept that very much*.”

*“*…*middle-class employed parents always think about the marriage of a girl child from her infant period itself. One of my friends stopped using cigars after the birth of the daughter to save money for her marriage. At the same time, he took an educational loan for his son for the higher education. He always said that daughter will go from the family to someone’s kitchen. But a son will always be with parents in their elder period and will get something to drink or eat to us till death*.”

### Schooling factors

The third domain constitutes the factors related to the education acquired from school. Since it is a strong influence on the CSEP, it carries nearly 6 items. Type of the school in which the participant studied (government/aided/unaided), method of commuting to school (by walk, by school bus, by private vehicle and by own vehicle), medium of instruction in the school (English or Malayalam), enrollment in the school meal program and opportunities for higher education after 10th standard (if they completed 10th standard) are included in the factors related to schooling. Poor children have the school meal as their major food in a day.

*“…Children from the employed parents prefer the unaided. English medium schools. They arrange school buses or other private vehicles as a method of commuting to school. Most of them have home tuitions. Poor children are going to school for getting the one time meal as part of school meal program. So they have an opportunity to take full meal at least once in a day…. Upper class parents sent their children to the institution with all amenities, which was situated in the cities. They can meet the hostel expenses of their children. They gave more preference to a good job.”*

### Childhood food

The fourth domain is related to childhood meal related factors. There is reasonably large amount of food eaten in a regular occasion of a usual childhood day. Even though the participant’s household had typical Kerala style breakfast food like idli, dosa or puttu, this domain includes both the frequency of meals in a day and the use of sugar in the household. Having full meals thrice in a day indicated that the household is well off.

*“…rich households have puttu, idly or dosa as breakfast. Poor children consider special breakfast as a luxury. Rich children got training in in-door games in those days.*”

### Childhood play

Fifth domain is childhood play related characteristics. The frequency of indoor games as well as the frequency of outdoor games in a week comes under the childhood play related factors. Financially well-off parents allow their children to play indoor games while poor and middle-class children play outdoor games usually.

“*We were playing thalapanthu kali and panthukali after completing our school periods, Boys from financially well off family had strong intention to play with us, but their parents didn’t like that. Parents of my friends are college lecturers. They trained and allowed him to play chess, and badminton. So most of the students from the elite families were likely to play indoor games.*”

### Household factors

The sixth domain is household-related factors. It is mainly related to the accessibility to home appliances, household facilities, and non-financial household assets. Household facilities include the presence of full time or part time domestic help. The non-financial household asset includes the roof of the house viz. thatched, tiled, concrete or mixed, the type of kitchen utensils used viz. steel, copper, bronze and aluminum (having steel utensils was considered luxury), and access to the vehicle (registered vehicle owned by their parents or other family members). The ownership of the household electric appliances like television, radio, refrigerator and land phone is included in the accessibility of home appliances related question.

“*Radio, was a luxury electric appliance in rural areas. Having bicycle was a big thing. I went to my uncle’s house, which located in city. I saw a black and white television in that house. Uncle was an assistant manager in a bank. His bank quarters was a concrete one. It was a two stored house.*”

### Use of health care facilities

**Figure 2:**
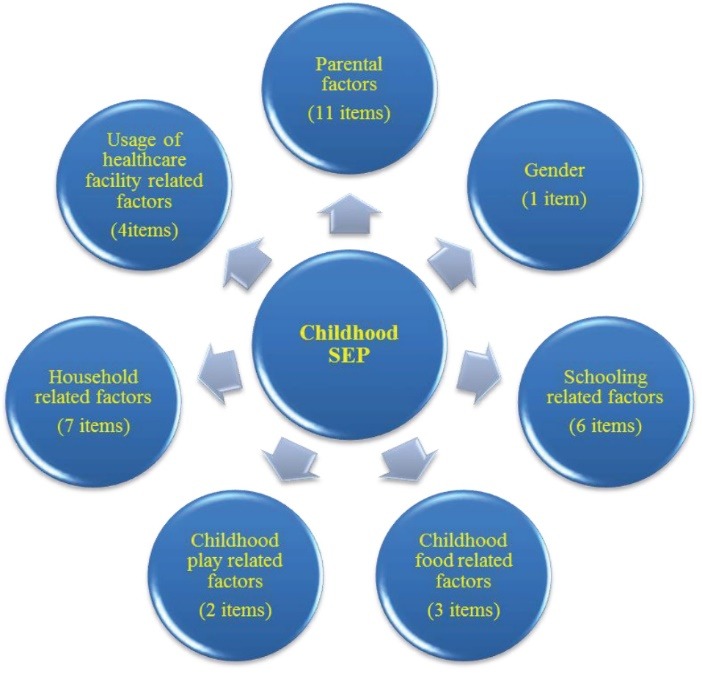
Domains of childhood socioeconomic position.

The last domain includes the factors related to the use of health care. It includes the history of sibling’s death (indicating the poor status of household), history of home delivery of participant’s mother, immunization to the self and siblings and the type of health care facility (either government or private) used during the time of childhood ailments. Home delivery was rare in the upper-class households. Recurrent infections for the children and child death were very common in poor households in that period (See Figure 2).

*“Death of children with unknown causes was very usual among the poor households. Still birth was very common. Rich households always preferred English (Allopathic) medicine to cure their illness. Government health system for the poor patients.”*

The textual analysis of the transcripts of in-depth interview extracted a list of 42 items. These 42 items were distributed under 7 domains.

### Content validity

By discarding those items of the questionnaire that were less related to the childhood SEP, the number of items decreased from 42 to 28. A 28-item questionnaire was there for validation analysis, after a cautious rejection of 14 items.

### Reliability - Internal consistency

The values of α and stratified α were 0.89 and 0.85, respectively. The value of α did not vary significantly with the elimination of any item. A detailed analysis of each item is shown in Table 2. This table consists of the items, with the corrected item to total correlation value >0.3. After doing the reliability analysis, the items reduced to 23 items questionnaire under 3 domains.

### Construct validity

The Kaiser-Meyer-Olkin (KMO) measure of the sampling adequacy was found to be 0.82, and Bartlett’s test concluded that the hypothesis of sphericity could be rejected (*P* < 0.05). Three factors are explaining 55% of the variance in the analysis (Table 3). After doing the factor analysis, we had an 11-item questionnaire to measure the CSEP. The name of the domains is renamed after observing the characteristics of variables under each factor.

The mean score of childhood SEP questionnaire in this population, in which it was developed, is 24.88 (+9.18) and ranging from 0 to 43.

### Description of the questionnaire 

**Figure 3:**
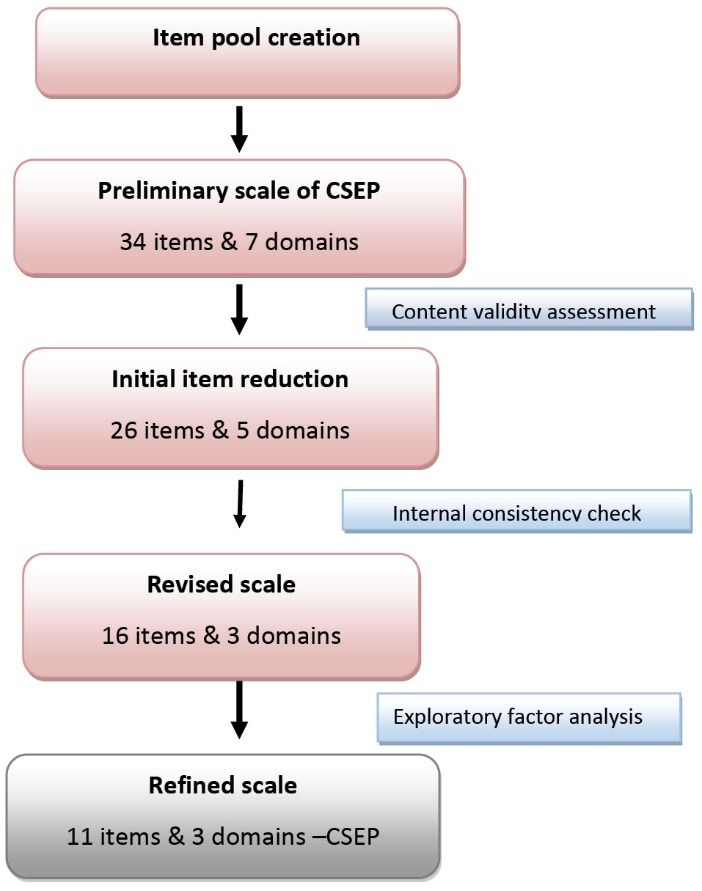
Questionnaire development process.

This is a self-administered questionnaire developed in the local language of Kerala (Malayalam) and aimed to use in noncommunicable disease epidemiological research. Responses of the questions in a mixed format. It included both yes/no and multiple choices. We used theoretical – participatory approach. In this approach, researchers randomly order the options under each question. We asked the participant and the members of expert panel to rank the options under each question. Respondent ranked the options under each question and compared the items to each other by placing them in order of their understanding about the SEP hierarchy that existed during their childhood. We provided equal intervals between successive ranks in each question. This ranking order is checked by an expert panel and a group of lay persons (between the age of 18-45 years). Each rank response carries a weightage points and should be blinded from the respondent during administration. It has a minimum score of 0 and maximum score of 43 (Figure 3).

## Discussion

CSEP questionnaire is a valid and reliable tool in public health research. The questionnaire consists of 11 items distributed across 3 domains. It consists of closed-end questions with options carrying different socioeconomic meanings. Scoring is done adding the scores of the individual item of the questionnaire. Exploratory factor analysis revealed a three-factor structure which indicates the multidimensionality of the construct CSEP. This finding is unique in the measurement of CSEP in public health research.

Most of the available CSEP tools used either the parent’s occupation (to represent the material background of the individual’s early life) as a single indicator or the parent’s education (to represent the intellectual background of the individual’s early life) as an additional indicator to represent the childhood SEP retrospectively. Retrospective measurement of parental occupation at a single age is a weak proxy for more complete information on SEP spanning the entire childhood period. It might be the good and easy to know indicators for those who are living in a highly urbanized and organized society of a developed country. In a society, more depends on agriculture and undergoing socioeconomic transition, these 2 indicators are becoming insufficient to measure the childhood SEP. Shortcomings of retrospective measures of childhood SEP can be empirically solved by increasing the number of indicators to measure the childhood SEP.

### Domains of childhood SEP

Value added through paternity – This domain is about the paternal role (father’s role) in the process of household decision making. The facilities enjoyed by the family members are decided by the father’s occupation. It is representing the existence of the patriarchal society. Father was the deciding person and the head of the household. According to theory of family buying decision, the single most consistent finding is that the father plays an instrumental role/ single decision maker (idea man) and the mother plays the expressive (emotional) role in family decision making.^9,14^ The status of single decision maker in the household depends upon the tangible resources (education, occupation and income).^12^ If the father has more tangible resources, then the mother is more likely to acquiesce and allow the father to make the decisions regarding household matters always or most of the time.^14^ It is based on the notion that single decision maker acts for the good of the entire household and it assumes that all household resources are allocated by a household head who represents the member’s taste and preference. Other theories in the studies of family sociology to substantiate the paternal dominance in family matters are Parson’s theory of social stratification and unitary model (common preference model). That theory considers family as the unit of solidarity and accepts the single breadwinner status of the father^14^ in a patriarchal society, which mostly existed in Kerala in the period of 1960-1980s. The method of commuting to school is a proxy measure of type and status of the school which participant attended. School meal program started to ensure the full meal one time in a day for the poor children. The enrolment in the school meal program of children says the status of the household. This indicator was predominant in many of the available measures of childhood SEP. The medium of instruction at school is important – rich households and those who are living in an urban setting have the access to schools which have English as medium of instruction (English medium schools are considered elitist in India). No Indian scaled measuring childhood SEP has so far included this aspect. The method of commuting to school is an indicator that directly says about the facilities available to a child in the family and particularly about the socio-economic position of the family. Having a school bus is a luxury for the rural setting. In 1970-1980s, television and refrigerator^14^ represent a luxurious style of living in Kerala. Caste also determined the individual’s accessibility to various facilities like education and occupation in a society where the remnants of caste discrimination existed.^14^ All the indicators of this domain are the mirror image of the typical societal structure of rural Kerala, which existed in the period of 1960–1970.

Maternal occupation - Doing a formal employment by a female family member was very less except in some socially forwarded families. The status of ‘housewife’ is a matter of prestige in most of the families in Kerala. In the early 1960s, most of the highly educated Malayalee (Keralite) women were doing the job of a teacher or clerk.^14^ In early 1970s, doing a formal job (a job with fixed salary) by a female member of the household and meeting the household expenditure by the earnings of that employed female family member were considered as a matter of shame and inferior status, indicative of socially disadvantaged position in Kerala society, where patriarchy had strong roots.^14-16^ The absence of any associated variables other than maternal occupation clearly indicates the poor role of women in household decision-making which was observed in most patriarchal societies.^17^

Parental education – Education is a frequently used indicator in most of the SEP scales used in life course epidemiology. The use of education as a childhood SEP indicator has its historical origins in the status domain of Weberian theory, and it attempts to capture the knowledge related assets of a person.^11,15,16^ It reflects material, intellectual, and other resources of the family originating in childhood, and is influenced by access to and performance in primary and secondary school and reaches its final attainment in young adulthood for most people. Access to education was decided by the SEP. Education was a luxury good for both women and socially disadvantaged castes in 1930-1960s.^17-20^ There have been considerable changes in educational opportunities for women over recent decades. Educated father would get a job easily compared to the less educated mother. Parent’s education decides their job status, exposure to the modern amenities. It is already universally recognized as a domain to measure childhood SEP.^15,16^

The logical sequence of the questions made easy to answer by the respondent. The average response time was 12 minutes in the cognitive interview. This duration is less than the maximum of 30 minutes recommended for studies where an interviewer applies the questionnaire. The diversity of the questionnaire made the tool more comprehensible. We added some memory clues and instructions for answering each type were provided. This made the individual to recollect the childhood memories easily.

The retrospective nature of childhood SEP questionnaire provides an ample opportunity to empirically examine theoretical life course models in the absence of complete data across the life course.^21^ Our composite measures of SEP rely on the assumption that SEP indicators are measured with the same precision across the lifespan. The potential misclassification issues related to a single indicator like father’s occupation is limited in this study. This study includes middle-aged people, questions relating to childhood were asked several decades after the event, although any recall bias hopefully was minimized by the use of memory clues and validating the reports with the cross-reference to the immediate caregiver to collect the retrospective information.

### Limitation

The questionnaire had not been validated extensively. It requires testing in the larger sample to make it more valid and robust. The main limitation is that, although measuring the same underlying concept, these indicators may be specific to the temporal and geographical context where they were developed and thus be difficult to compare across studies. Most important was the use of self-reported data, which may have produced misclassification of exposure status. Reliance on self-reports may have introduced reporting bias.

## Conclusion

The CSEP questionnaire developed in this study to assess the CSEP can be considered valid for its application in the population studies. Validity and reliability results show that this can be a good instrument to assess the CSEP of younger adults of Kerala.

## Ethical approval

The research was approved by the Ethics Committee of Sree Chitra Tirunal Institute for Medical Science & Technology, Trivandrum, Kerala (SCTIMST/IEC/668). Participants provided consent that the interviews could be recorded, analyzed and published the reports later.

## Competing interests

The authors declare that they have no competing interests.

## Funding

The study was funded by the student research grant of Kerala State Council for Science, Technology and Environment, Kerala, India.

## Authors’ contributions

UVS did the conceptualization and drafting of the manuscript. VRK and ATN provided input into the study design, provided intellectual input to the manuscript and approved the final version of the manuscript.

## Acknowledgments

Dr Mala Ramanathan, Professor, Achutha Menon center for Health Science Studies helped the researchers to teach and streamline the process at each steps of methods of this study.

**Table 1:** Study sample characteristics

**Characteristics**	**Proportion (N = 200)**
Gender	
Male	109
Age group	
25-35 years	84
36-45 years	116
Area	
Rural	111
Urban	99
Socioeconomic status	
Upper	49
Middle	72
Lower	79
Employment status	
Salaried employed	118
Unofficial paid jobs	82
Region	
Kannur (North)	65
Palakkad (Central)	68
Trivandrum (South)	67
Marital status	
Married	149
Unmarried	49
Divorced/separated	2

**Table 2:** Reliability assessment: Item to total correlation of child hood SEP questionnaire

	**Variables**	**Crohnbach’s alpha value if one Item deleted**
**D1**	**Parental Occupation **	
1	Permanent income for father	0.67
2	Category of father’s job– Private/Government	0.51
3	Occupation of father	0.81
4	Permanent income for mother	0.67
5	Category of mother’s job – Private/Government	0.60
6	Occupation of mother	0.72
**D 2**	**Parental Education **	
7	Education of father	0.80
8	Education of mother	0.83
**D 3**	**Schooling **	
9	Medium of instruction at school	0.70
10	Enrolment in the school meal program	-0.58
11	Method of commuting to school	0.74
**D 4**	**Household Assets**	
12	Having fulltime domestic help	0.53
13	Electronic appliances	0.77
14	Type of utensils	0.51
15	Having access to vehicles	0.59
16	Roof of the house	0.80

Note: Alpha value (Kline’s value) is >0.50 for the sample size 200. Now it is 16 variables distributed across 4 domains.

**Table 3:** Exploratory factor analysis: Result for childhood SEP questionnaire

**Items**	**Name of the domain**	**Factor 1**	**Factor 2**	**Factor 3**
Electric appliances	Value added through paternity	0.797		
Roof of the house	Value added through paternity	0.754		
Category of Father occupation	Value added through paternity	0.681		
Type of utensils	Value added through paternity	0.673		
Vehicle ownership	Value added through paternity	0.606		
Method of commuting to school	Value added through paternity	0.603		
Enrolment in School meal program	Value added through paternity	-0.553		
Had full time domestic help in the home	Value added through paternity	0.526		
Medium of learning in children’s school	Value added through paternity	0.449		
Mother – nature of the job	Maternal occupation		0.987	
Mother occupation categories	Maternal occupation		0.825	
Mother education	Parental education			0.849
Father education	Parental education			0.755

Extraction method: Maximum likelihood method with direct oblimin rotation. Loadings of the rotation matrix presented.
